# Classification of Tea Leaves Based on Fluorescence Imaging and Convolutional Neural Networks

**DOI:** 10.3390/s22207764

**Published:** 2022-10-13

**Authors:** Kaihua Wei, Bojian Chen, Zejian Li, Dongmei Chen, Guangyu Liu, Hongze Lin, Baihua Zhang

**Affiliations:** 1 School of Automation, Hangzhou Dianzi University, Hangzhou 310018, China; 2 Digital Economy Research Institute, Hangzhou Dianzi University, Hangzhou 310018, China; 3 Zhejiang Key Laboratory of Design and Intelligence and Digital Creativity, College of Computer Science and Technology, Zhejiang University, Hangzhou 310027, China; 4 Shangyu Institute of Science and Engineering, Hangzhou Dianzi University, Shaoxing 312000, China; 5 School of Artificial Intelligence, Wenzhou Polytechnic, Wenzhou 325000, China

**Keywords:** tea, classification, fluorescence imaging, convolutional neural network (CNN), deep learning, LED-induced fluorescence, VGG, ResNet

## Abstract

The development of the smartphone and computer vision technique provides customers with a convenient approach to identify tea species, as well as qualities. However, the prediction model may not behave robustly due to changes in illumination conditions. Fluorescence imaging can induce the fluorescence signal from typical components, and thus may improve the prediction accuracy. In this paper, a tea classification method based on fluorescence imaging and convolutional neural networks (CNN) is proposed. Ultra-violet (UV) LEDs with a central wavelength of 370 nm were utilized to induce the fluorescence of tea samples so that the fluorescence images could be captured. Five kinds of tea were included and pre-processed. Two CNN-based classification models, e.g., the VGG16 and ResNet-34, were utilized for model training. Images captured under the conventional fluorescent lamp were also tested for comparison. The results show that the accuracy of the classification model based on fluorescence images is better than those based on the white-light illumination images, and the performance of the VGG16 model is better than the ResNet-34 model in our case. The classification accuracy of fluorescence images reached 97.5%, which proves that the LED-induced fluorescence imaging technique is promising to use in our daily life.

## 1. Introduction

As one of the major beverages worldwide, tea plays a vital role in our daily life. It is widely believed that drinking tea contributes to freshness of spirit, enhances thinking and memory [1], delays the formation of lipid plaques in the vascular intima, prevents arteriosclerosis and high blood pressure, etc. These functions are attributed to the abundant nutrition that tea contains, including protein, amino acids, carbohydrates, vitamins, inorganics, polyphenols, and other trace components that are beneficial to the human body [2,3,4]. One of the most famous components is Epigallocatechin gallate (EGCG), which is a bioactive polyphenol in green tea and has received extensive attention [5]. Recently, with the increase in labor cost and inflation, the price of tea keeps rising, leading to the phenomenon of shoddy products appearing in the market. However, it is difficult for customers to tell apart high-level tea from the lower with naked eyes. Therefore, a convenient method that can help consumers classify tea species, as well as qualities, is of great importance and in great demand.

The traditional evaluation of tea is carried out manually by experts who grade the tea samples according to their aroma, color, and shape [6]. The evaluation results are non-repetitive, and are easily affected by the physical and mental states of the evaluators. Meanwhile, this process is time-consuming and expensive, which prevent its use by customers. Methods e.g., Vis-NIR spectroscopy [7,8,9,10], Fourier transformed infrared spectroscopy [11,12], laser-induced breakdown spectroscopy [13], chemical analysis [14], X-ray fluorescence spectroscopy [15], electronic nose [16], and liquid chromatography [17] have all been applied to tea classification. Although the classification abilities of these methods are good, drawbacks of high costs and large volumes of the experimental systems limit their utilization to laboratory experiments.

In recent years, image classification technology based on computer vision has been developing rapidly. This technology can be easily transferred to platforms e.g., the smartphone, which owns a strong calculation ability and high-resolution complementary metal oxide semiconductor (CMOS) image sensor, and has already been applied to the fields of food authentication [18], tea leaf diseases detection [19], etc. Compared with conventional digital image processing methods, the computer vision shows characteristics of higher stability and precision [20], and has made preliminary progress on tea leaves [21,22,23,24]. Bakhshipour et al. extracted and evaluated 18 color features, 13 gray image texture features, and 52 wavelet texture features for black tea. They employed correlation-based feature selection and principal component analysis (PCA) to select the most significant features. The network with seven inputs, ten hidden layers, and four outputs was finally developed, and the highest accuracy rate achieved was 96.25% [22]. However, the methods above are mainly based on the color features [6,22,25,26], texture features [6,22,25], and wavelet features [22,25], which requires the images to be filled with tea leaves. Furthermore, as the illuminant condition may vary from case to case, the prediction models built may lose their robustness. The shape features are also employed for tea classification. However, with only the shape parameters, e.g., leaf width, leaf length, and leaf area, it is hard to build a robust and precise prediction model [9]. Hyperspectral imaging can achieve a very-high prediction accuracy. Yet, the device is too expensive for customers, and the methods used to build the prediction model are based on averaged spectra, not images [27,28,29]. Using the deep learning method, Kamrul et al. deployed three models: VGG16, sequence model, and Faster R-CNN to classify fresh tea leaves. The final average accuracies reached 95.23%, 92.23%, and 96.28%, of sequence model, VGG16 model, and Faster R-CNN model, respectively [30]. Latha et al. applied the convolutional neural network (CNN) model, which includes one input layer, four convolutional layers, and two fully connected layers, for tea diseases detection, and achieved a correct identification accuracy of 94.45% [31]. Puja et al. applied the Grad-CAM technique for the explanation of tea-leaf classification, whose targets were fresh tea leaves [32].

Fluorescence spectroscopy, especially laser-induced fluorescence (LIF) spectroscopy [33], has the advantages of simple operation, high sensitivity, low cost, as well as fast response speed, and has been widely used in the field of food detection [34,35]. Nowadays, with the increasing light intensity and its easy operation characteristic, LEDs are extensively used as an alternative excitation source to induce fluorescence signals, referred to as LED-induced spectroscopy [36,37,38,39,40]. Silva et al. used light-emitting diodes (LEDs) and CMOS array sensors to capture the fluorescence spectrum in a diluted oil sample, and classified the vegetable oil through a three-layer artificial neural network (ANN) [41]. Lin et al. established a fluorescence system employing seven excitation LEDs with wavelengths ranging from ultraviolet to blue to induce fluorescence signals from samples, and combined those with a convolutional neural network to classify tea leaves. The results showed that the accuracy of tea classification is significantly improved compared with traditional methods, e.g., PCA combined with k-nearest neighbor [42]. Different from the traditional computer vision method, fluorescence-based methods are conducted under a dark environment, which prevents the influence of surrounding illumination. Meanwhile, the light source used for excitation is uniform from case to case, and the wavelength is selected according to the fluorochrome, thus improving the robustness of the prediction model.

In this paper, a method for the classification of tea based on the fluorescence imaging and deep learning is proposed. Due to the different pigment contents among different varieties of tea, the fluorescence imaging was used to improve the recognition degree of tea characteristics. Five tea samples with similar appearances were tested using a colorful CMOS camera to capture the fluorescence images illuminated by LEDs with a central wavelength of 370 nm, as well as images illuminated by white light for comparison. The models were trained by deploying two deep learning classification models, the VGG16 and ResNet-34, respectively. All of the models were trained on manually labeled data sets. The two groups of images were processed with the same color feature and imported into the model for training. This paper aims to address three questions: (1) Will fluorescence imaging improve the prediction accuracy; (2) do all three channels of the figure (RGB) contribute to the prediction model, or does just one single channel contribute the majority; (3) does the channel selection improves the prediction accuracy compared with the results obtained from monocolor images. The results show that the classification accuracy of the fluorescence images of tea is better than those illuminated by the white light and prove that the 370 nm wavelength LED irradiation is helpful to the classification ability.

## 2. Materials and Methods

### 2.1. Image Acquisition

**Figure 1:**
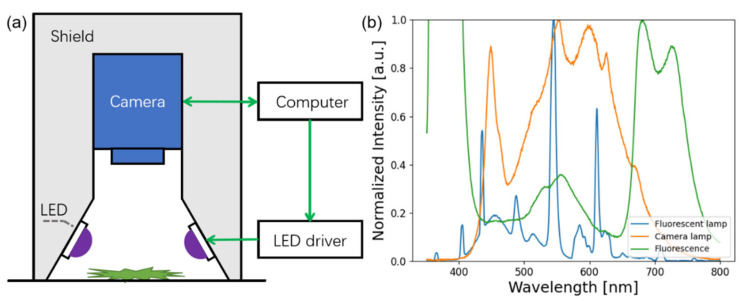
(**a**) Schematic diagram of fluorescence imaging device. The LEDs, LED driver, and shield were removed when taking images under white light. (**b**) Spectra of fluorescent lamp (blue line), smartphone camera lamp (orange line), and tea fluorescence spectrum under excitation of 370 nm LED (green line). Spectra were normalized to their maximum amplitude after 450 nm, respectively.

The system to obtain the fluorescence images is depicted in Figure 1a, which mainly consisted of a circular LED lamp, a shield, and a colorful CMOS camera (ASI120MC-S, ZWO Inc., Suzhou, China). The LED lamp contained around 100 small LEDs facing to the central with an incident angle of 60°. The central wavelength was chosen as 370 nm, because this wavelength band can induce the fluorescence not only from the chlorophyll *a* in the red and far-red band, but also from the tea polyphenols in the yellow band [42]. Figure 1b shows the spectra of the tea fluorescence signal, the fluorescent lamp, and a smartphone camera lamp, respectively. All of them were normalized to their maximum amplitude after 450 nm, respectively. The spectra of the fluorescent lamp and the smartphone camera lamp are quite different, showing that the illumination of room’s light may vary from case to case and thus may influence the prediction ability of models if the images were taken by the cameras of consumers. The camera of the system was placed above the center of the circular LED lamp at a distance of 12 cm to the tea samples, and its focal length was tuned to obtain a clear figure. The tea leave samples were placed on a black flannel, which had no inelastic response to the UV light. To ensure reproducibility, the CMOS camera and LEDs were installed tightly to maintain their position. The images taken under white light were taken under illumination of a white fluorescent lamp, removing the circular LED lamp, the LED drive, and the shield.

### 2.2. Sample

**Figure 2:**
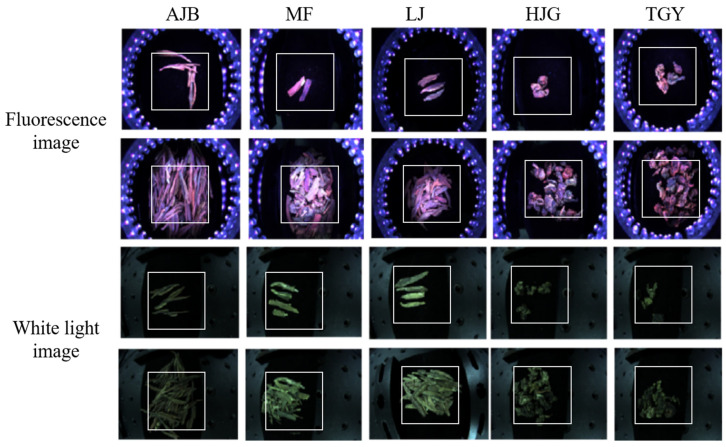
Fluorescence images and white-light images of five different kinds of tea. The ROIs are squared in white boxes. AJB, MF, LJ, HJG, and TGY stand for the Anjibai tea, Maofeng tea, West Lake Longjing tea, Huangjingui tea, and Tieguanyin tea, respectively.

Five tea samples were purchased from a local market, namely, the Anjibai tea (AJB), the Maofeng tea (MF), the West Lake Longjing tea (LJ), the Huangjingui tea (HJG), and the Tieguanyin tea (TGY). The LJ, AJB, and MF teas belong to the green tea group, while HJG and TGY belong to the oolong tea group. From each tea, 76 fluorescence images and 76 white-light images were taken by inserting the samples to the field of view of the camera, as depicted in Figure 1a. Among them, 60 belong to the initial training set, and the remaining 16 images belong to the test set. Figure 2 shows typical images of the five different tea species under the two illumination conditions. The tea images recorded may include clusters or just a few numbers of tea leaves to enrich the diversity. The fluorescence images have relatively high values in the R channel, while the white-light images have relatively high values in the G channel, reflecting the fluorescence and absorption characteristics of chlorophyll a, respectively, which is the most abundant component in tea leaves.

### 2.3. Image Preprocessing

#### 2.3.1. Region Extraction

In order to remove the background as well as the influence of LEDs on the tea classification results, the region of interest (ROI) was extracted from all original images, as shown in Figure 2 with white boxes. The size of the ROI was 660 × 660 pixels.

#### 2.3.2. Data Augmentation

**Figure 3:**
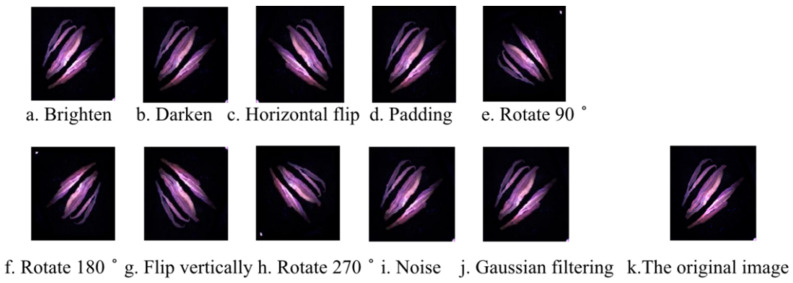
Methods and results of data augmentation transformation of different methods (**a**–**j**), and the original image (**k**).

The size of the data set affects the performance of the model. When the data set is not large enough, overfitting results. Therefore, it is necessary to increase the amount of data for deep learning. Due to the fact that the amount of the data collected is small and there is no public data set of tea currently, more images were generated through the ten data augmentation methods, i.e., brighten, darken, horizontal flip, vertical flip, padding, noise, gaussian filtering, rotate 90°, 180°, and 270°. Figure 3k is a fluorescence image after region extraction. Figure 3a–j shows a series of transformation results. After data augmentation, both the training set of the fluorescence image data set and the white-light image data set were expanded from 300 to 3300.

#### 2.3.3. Deep Learning Model

##### VGG16 Model

**Figure 4:**
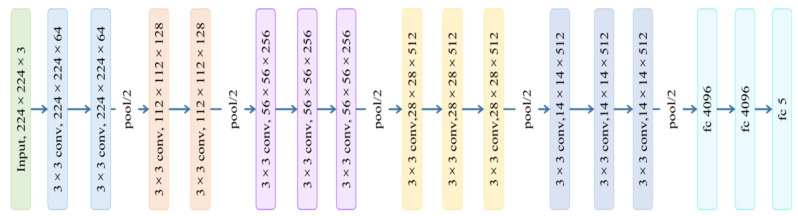
VGG16 model structure diagram. *conv* stands for convolutional layer. fc stands for fully connected layer.

VGGNet is a convolutional neural network model proposed by Simonyan and Zisserman [43]. It studies the connection between the depth and performance of neural networks. VGGNet builds a deep convolutional neural network by repeatedly using a 3 × 3 convolution kernel and a 2 × 2 maximum pooling layer, which greatly increases the depth of the network. Compared with convolution kernels of other scales, 3 × 3 convolution has higher computational density and is more efficient. The model structure of VGG16 is as shown in Figure 4.

It can be seen from Figure 4 that the VGG16 network model contains five large convolution modules, and each large convolution module has two or three convolution layers. The model contains a total of 13 convolution layers, and each large convolution module is followed by a maximum pooling layer to reduce the size of the picture by half. A 3 × 3 convolution kernel is used in the VGG16 network, because the concatenation effect of two 3 × 3 convolution kernels is similar to a 5 × 5 convolution kernel, and the convolution effect of three 3 × 3 convolution kernels is similar to a 7 × 7 convolution kernel. By using smaller convolution kernels, the model parameters are significantly reduced. Furthermore, it contributes more nonlinear changes, which can provide the network with stronger feature-learning capabilities. The model is connected to three fully connected layers at the end; the former two fully connected layers have 4096 channels, and the latter layer is used for classification.

##### ResNet-34 Model

**Figure 5:**
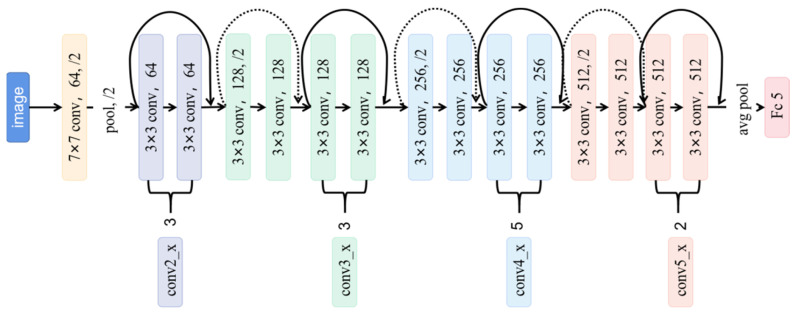
ResNet-34 model structure diagram.

The deep residual network Resnet structure was proposed by He Kaiming et al. [44] Its design aim was to solve the “degradation” problem that occurs when the network deepens in the convolutional neural network. In order to solve this problem, an identity shortcut connection structure is introduced. For a stacked layer structure, the learned feature can be expressed as:

H(*x*) = F(*x*) + *x* (1)

where *x* is the input and F(*x*) is the residual. When the residual is 0, the accumulation layer only performs identity mapping at this time, and the network performance will not decrease. In fact, the residual will not be 0, which will also make the stacked layer learn new features based on the input features, and in this way improves the performance. The classic ResNet networks include ResNet-18, ResNet-34, ResNet-50, ResNet-100, etc. This study used the ResNet-34 model. The specific network structure is shown in Figure 5. The curve in the figure represents a residual unit.

##### Transfer Learning

Transfer learning refers to a method in which a model is trained on the original domain and then the training results are applied to the target domain. This study used the weights trained by the above two models in the ImageNet data set and transferred them to this study to classify the fluorescence data set and the white-light data set, observe the results of these two models on the tea classification project, and identify the best model structure.

#### 2.3.4. RGB to Grayscale Converting

Colorful images were converted into mono-color images according to Equation (2):

(2) $$Grayscale_{pix}=0.2989\times R_{pix}+0.5870\times G_{pix}+0.1140\times B_{pix}$$

where *Grayscale_pix_*, *R_pix_*, *G_pix_*, and *B_pix_* are the grayscale, R channel, G channel, and B channel value of each pixel of a processed figure, respectively.

### 2.4. Evaluation Method

The widely used evaluation indexes, e.g., accuracy, precision, and recall, were employed. Their definitions are as follows:

(3) $$Accuracy=\frac{TP+TN}{TP+TN+FP+FN}$$

(4) $$Precision=\frac{TP}{TP+FP}$$

(5) $$Recall=\frac{TP}{TP+FN}$$

where *TP* represents true positive, *FP* represents false positive; *TN* represents true negative, and *FN* represents false negative. When dealing with multi-classification, the macro-average method was used to obtain the precision and recall of all five kinds of tea.

### 2.5. Flowchart of the Proposed Methodology

**Figure 6:**
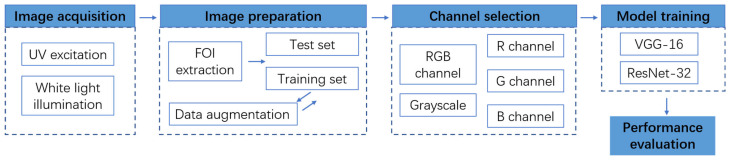
Flowchart of the proposed tea classification methodology.

Figure 6 shows the flowchart of the proposed tea classification methodology. The images were collected under two conditions, i.e., the UV excitation condition and the white-light illumination. In total, 380 images of each condition were acquired, and the FOIs were extracted. These samples were then separated into the training set and test set. The training set contained 300 samples and would be later expanded to 3300 through ten data augmentation methods, as described in Section 2.3.2. The test set contained 80 samples. Before model training, channels of the sample were selected or transformed. Images for training included single-channel images of the R, G, and B channels as well as gray-scaled images. Multi-channel images, e.g., the RGB images, also served as the input. These images were put into deep learning models of VGG16 and ResNet-32, and their performances were evaluated with accuracy, confusion matrix, and, more importantly, with comparison among channel selection methods and between image acquisition methods.

## 3. Results

### 3.1. Analysis of Fluorescence Images of Tea

One purpose of this paper is to explore whether all three channels of the figure contribute to the prediction model, or whether just one single channel contributes the majority. Therefore, the experiment performed single-channel (R, G, B) extraction from each image, and at the same time, gray-scale processing was performed to obtain a mono-color image. The results were compared with the results of the RGB figures.

**Figure 7:**
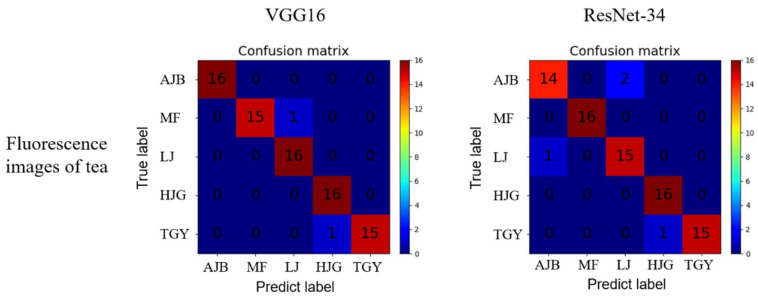
Confusion matrix of fluorescence RGB images of tea.

Table 1 shows the accuracy of the tea fluorescence images of the test data set, using the extractions of each channel, the grayscale figure, and the RGB figure. The VGG16 and ResNet-34 models are utilized for training with the same learning rate and batch size. It can be observed that the result of single-channel (R, G, B, and grayscale) tea classification is not decent, achieving a maximum accuracy of merely 80%. On the contrary, the result of RGB tea fluorescence image classification is significantly higher than the single-channel data set, as the accuracies of both models are above 95%. The prediction result of the VGG16 model is better than that of ResNet-34 model, reaching a test-set prediction result of 97.5%. Figure 7 shows the confusion matrix of both models using RGB images. When using the VGG16 model, only one MF sample was mistakenly classified into LJ, and only one TGY sample was mistakenly classified into HJG. When using the ResNet-34 model, two AJB samples were classified into LJ, and one LJ sample was classified into AJB. The classification results of HJG and TGY were the same as VGG16.

### 3.2. Analysis of White-Light Images of Tea

To check whether fluorescence imaging would improve classification accuracy, tea images obtained under the illumination of an ordinary white fluorescent lamp were also trained and tested. The pre-processing methods and the models used were kept the same as used for the fluorescence images for comparison.

**Figure 8:**
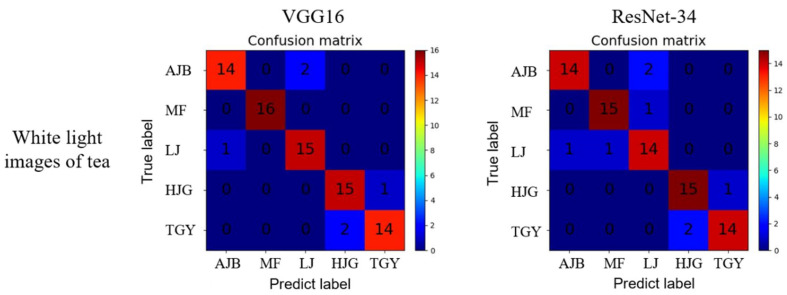
Confusion matrix of white-light RGB images of tea.

Table 2 shows the accuracies of the white-light images. It can be found that the classification results of white-light tea are similar to the fluorescence classification results. The four single-channel image classification results of R, G, B, and grayscale achieved a maximum accuracy of 77.5%, which was much lower than the accuracies achieved by the RGB figure as well. Both models trained based on the RGB three-channel image reached classification accuracies above 90%. Figure 8 shows the confusion matrix of both models using RGB images. When using the VGG16 model, two AJB samples were identified to LJ, and one LJ sample was identified to AJB. One HJG sample was identified to TGY, and two of TGY samples were identified to HJG. When using the ResNet-34 model, besides the misclassification of VGG16 mentioned above, one MF sample was identified to LJ.

### 3.3. Comparism of RGB-Image-Based Training Results

Table 3 shows the accuracies, precisions, and recalls of the two datasets and the two training models. It can be observed that with the same dataset, VGG16 can always obtain a better performance in accuracy, precision, and recall than ResNet-34, with an increase of around 2 percent. Comparing the results of the same deep learning modal but different dataset, the fluorescence images achieve better performance in accuracy, precision, and recall, with an increase of around 5 percent.

## 4. Discussion

By comparing the training results, it can be found that the classification results of fluorescence images are better than those of white-light images, with the highest classification accuracy reaching 97.5%. No misclassification between tea types was observed. The AJB and LJ and the HJG and TGY are the two categories that raised high classification error rates, which may attribute to their high similarity in shape. However, when dealing with fluorescence images, these errors were much reduced, which further shows that fluorescence imaging is helpful to improve the feature extraction ability of the model and improve the accuracy of tea classification.

In the results of the fluorescence images, the B channel shows slightly higher accuracy than other single-channel results when using the VGG16. Meanwhile, when using the ResNet-34, it is the G channel that behaves better. The same phenomenon happens in the results of white-light illumination. The accuracies of single-channel images are much lower than the RGB images in both cases. Thus, all three channels contribute to the prediction model, and no single channel that contributes the majority has been found. This may also be due to the fact that the single-channel images contribute only the shape features, while the RGB images contribute to both the shape and fluorescence spectral features.

By comparing the accuracy of grayscale images with the other single-channel images, it can be seen that the results of the grayscale images are always the worst. Thus, the channel selection process, e.g., by coating different filters on the pixels of CMOS in our case, can raise the prediction accuracy, and can be further improved if more passing bands are employed and optimized.

It can be seen from the results that the VGG16 network model is better than the ResNet-34 network model for the tea data set as a whole in our case.

## 5. Conclusions

In this paper, a tea classification method based on fluorescence imaging and deep learning was proposed. LEDs with wavelength of 370 nm were chosen as the light source to induce the fluorescence of the tea samples. Fluorescence images were obtained by a CMOS camera. Two deep learning networks, the VGG16 and ResNet-34, were employed to train the model. Data sets included the RGB figures, single-channel figures, and the grayscale figures. Images taken with illumination of conventional fluorescent lamp were also collected for comparison.

The results show that the VGG16 network model performs better than the ResNet-34 network; meanwhile, the time that the VGG16 takes to build the model is also longer. Models built based on RGB figures were better than those built based on single-channel figures, including the grayscale figures. For fluorescence images, this implies that though fluorescence signals from tea leaves are mainly in the red channel, the blue and green channel fluorescence signals also contribute to shape their characteristics. Comparing models based on fluorescence images with those obtained under white light, the former performs slightly better. This advantage may get strengthen when the model trained based on fluorescent lamp illumination is applied to samples taken under the illumination of other light sources, e.g., the light from a camera lamp or the sun. Still, the results obtained prove the feasibility to employ LED-induced fluorescence imaging for tea classification, and is promising to be used with smartphones in the future.

As can be observed from the design of the apparatus, when using this technology in real-life by replacing the camera with a smart phone camera, the additional UV LED device is still required. The light sources of the smart phone, e.g., the flashlamp and the light from the screen, can induce the fluorescence of chlorophyll using the blue band light at around 450 nm. However, this band is not short enough to induce fluorescence signals from components such as flavonoid, and thus would decrease the prediction ability of the model when employed as the excitation light source. Future work will be carried out in two aspects. The first aspect will be focus on minimizing or removing the UV LED device, including developing a LED-modulation method to reduce the ambient light interference in case where there is no shield. The second aspect will be focus on employing more excitation LEDs to induce more fluorescence signals from tea leaves, so that the method can be applied to the tea leaves’ adulteration condition, where the species of each individual leaf can be classified.

## Figures and Tables

**Table 1:** The classification accuracy of fluorescence images.

Channel for Training	Learning Rate	Batch Size	VGG16	ResNet-34
			Accuracy (%)	Accuracy (%)
R	10^−3^	32	72.5	70
G			72.5	80
B			77.5	72.5
Grayscale			72.5	70
RGB			97.5	95

**Table 2:** The classification accuracy of tea under white-light illumination.

Channel for Training	Learning Rate	Batch Size	VGG16	ResNet-34
			Accuracy (%)	Accuracy (%)
R	10^−3^	32	77.5	75
G			80	77.5
B			72.5	77.5
Grayscale			70	72.5
RGB			92.5	90

**Table 3:** RGB-image-based Training results.

Dataset	Deep Learning Model	Accuracy (%)	Precision (%)	Recall (%)
Fluorescence Images	VGG16	97.5	97.6	97.5
	ResNet-34	95.0	95.1	95.0
White-Light Images	VGG16	92.5	91.7	92.5
	ResNet-34	90.0	90.2	90.0

## Data Availability

Data are available on request from the corresponding author.
